# Supplementary material for: The Distribution of Available Prevention and Management Interventions for Fetal Alcohol Spectrum Disorder (2007 to 2017): Implications for Collaborative Actions
Source: Int J Environ Res Public Health. 2019 Jun 25;16(12):2244. doi: 10.3390/ijerph16122244 (PMC6617245; doi:10.3390/ijerph16122244)
Supplement: Supplementary file 1 [file ijerph-16-02244-s001.pdf]

Supplementary Materials

Table S1. Quality assessment results for included studies.

Author	Selection Bias	Design	Cofounders	Blinding	Data Collection Methods	Withdrawals and Dropouts
			**Prevention Intervention**			
**Clinical**						
Mwansa-Kambafwile., et al., 2011	Moderate	Weak	Weak	N/A	Moderate	Weak
Joya et al., 2016	Moderate	Strong	Strong	Moderate	Strong	Strong
Velasquez et al., 2010	Moderate	Strong	Strong	Moderate	Strong	Moderate
Payne et al., 2011	Moderate	Weak	N/A	N/A	Strong	Weak
**Educational**						
Boulter, 2007	Moderate	Weak	N/A	N/A	Moderate	Weak
LaChausse, 2008	Strong	Weak	Strong	Moderate	Strong	Strong
**Social**						
De Vries et al., 2013	Moderate	Moderate	N/A	N/A	Strong	Moderate
Montag et al., 2015	Moderate	Strong	Strong	Moderate	Strong	Strong
May et al., 2103	Moderate	Moderate	N/A	N/A	Strong	Moderate
Wilton et al., 2013	Moderate	Strong	Strong	Moderate	Strong	Moderate
Rasmussen et al., 2012	Weak	Moderate	N/A	N/A	Strong	N/A
Floyd et al., 2007	Weak	Strong	Strong	Moderate	Strong	Moderate
Hanson et al., 2012	Weak	Weak	N/A	N/A	Strong	Weak
Hanson et al., 2017	Weak	Weak	N/A	N/A	Strong	Weak
Letourneau et al., 2017	Weak	Weak	N/A	N/A	Strong	Strong
O'Connor and Whaley, 2007	Moderate	Strong	Strong	Moderate	Strong	Moderate
Hanson et al., 2013	Weak	Weak	N/A	N/A	Strong	weak
Farrell-Carnahan et al., 2013	Weak	Moderate	N/A	N/A	Strong	Moderate
France et al., 2014	Weak	Strong	Strong	Moderate	Strong	Weak
Dresser et al., 2011	Weak	Weak	Strong	Moderate	Moderate	Weak
Chersich et al., 2012	Weak	Weak	N/A	N/A	Strong	Strong
Tenkku et al., 2011	Weak	Weak	Strong	Moderate	Strong	Moderate
			**Management interventions**			
**Clinical**						
Nguyen et al., 2016	Strong	Strong	Strong	Strong	Strong	Strong
Zarnegar et al., 2016	Weak	Weak	N/A	N/A	Strong	Moderate
Wozniak et al., 2015	Moderate	Strong	Strong	Strong	Strong	Strong

Wozniak et al., 2013	Moderate	Strong	Strong	Strong	Strong	strong
Wilczynski et al., 2015	Weak	Weak	N/A	N/A	Strong	Strong
Yazdani et al., 2009	Moderate	Weak	Strong	N/A	Strong	Weak
Kable et al., 2015	Moderate	Strong	Strong	Moderate	Strong	Weak
Connolly et al., 2016	Moderate	Weak	N/A	N/A	Strong	N/A
Nash et al., 2015	Weak	Strong	Strong	Strong	Strong	Strong
**Educational**						
Coles et al., 2009	Moderate	Strong	Strong	Moderate	Strong	Strong
Kerns et al., 2010	Weak	Weak	N/A	N/A	Strong	Strong
Jirikowic et al., 2016	Moderate	Strong	Strong	Moderate	Strong	Strong
Adnams et al., 2007	Moderate	Strong	Strong	Moderate	Strong	Strong
Kable et al., 2007	Moderate	Weak	Strong	Moderate	Strong	Strong
Kable et al., 2016	Moderate	Strong	Strong	Moderate	Strong	Strong
McCoy et al., 2015	Moderate	Weak	N/A	N/A	Strong	Weak
Kerns et al., 2017	Weak	Weak	N/A	N/A	Strong	Moderate
Coles et al., 2015	Moderate	Strong	strong	Moderate	Strong	Strong
Wells et al., 2012	Moderate	Strong	Strong	Moderate	Strong	Strong
Keil et al., 2010	Weak	Strong	Strong	Moderate	Strong	Strong
O'Connor et al., 2016	Weak	Strong	Strong	Moderate	Strong	Strong
Schonfeld et al., 2009	Weak	Strong	Strong	Moderate	Strong	Weak
**Social**						
Leenaars et al., 2012	Moderate	Moderate	N/A	N/A	Moderate	N/A
O'Connor et al., 2012	Moderate	Strong	Strong	Moderate	Strong	Moderate
Petrenko et al., 2017	Moderate	Strong	Strong	Moderate	Strong	Weak
Reid et al., 2017	Moderate	Weak	N/A	N/A	Strong	Moderate
Petrenko et al., 2017	Moderate	Strong	Strong	Moderate	Strong	Strong
Millians and Coles	Moderate	Weak	N/A	N/A	Strong	N/A
Kable et al., 2015	Weak	Strong	Strong	Moderate	Strong	Strong
Pomeroy and Parrish, 2013	Moderate	Weak	N/A	N/A	Strong	Moderate
Hanlon-Dearman et al.,2017	Moderate	Strong	Strong	Moderate	Strong	Moderate

Confounders were not assessed for one-group studies, blinding was not assessed for one-group studies and case study and withdrawals/dropouts were not assessed for a case or retrospective studies

Table S2. Data extracted from the included studies.

Author	Country	Study Design	Sample Size and Population	Approach and Follow up	Result
**Prevention Intervention**					
**Clinical**					
Mwansa-Kambafwile et al., 2011	South Africa	Pre-post-test design	86 service providers with 23 control group	Training on the importance of a brief interview in the treatment of alcohol problems, the importance of contraception for AEP, and ways to improve the referral system. No follow-up reported	The finding showed the effectiveness of a brief motivational interview in building the capacity of service providers to be able to prevent and manage FASD.
Joya et al., 2016	Spain	Randomized control trial	168 pregnant women attending antenatal visit were included in the study	Some participants received single session motivational Intervention and other educational control condition. Mothers completed the Ethanol Timeline Follow-back for Ethanol consumption from the time of study enrolment until delivery. Follow up reported at 2nd and 3rd trimester of the pregnancy.	Single-session MI helped in decreasing alcohol consumption during pregnancy.
Velasquez et al., 2010	USA	Randomized control trial	830 women were randomized to either the motivational counselling (information plus counselling; IPC) group (n = 416) or the information only (IO) group (n = 414).	Each participant was offered four counseling sessions, plus the visit to a contraception provider, delivered over the course of 10 weeks. Women were also given informational brochures on alcohol, health, and available birth control methods. Follow-up was reported at 9 months.	Project CHOICES demonstrated the efficacy of a dual-focused adaptation of motivational interviewing to reduce the risk for AEP.
Payne et al., 2011	Australia	Survey	1483 health professionals	Educational resources were delivered to health professionals, Follow-up reported at 6 months.	Increased health professionals' knowledge, changed in attitudes and practice about FASD, and the advice they give to pregnant women about alcohol consumption.
**Educational**					
Boulter, 2007	USA	Non-randomized controlled trial	642 students (267 males, 375 females) from five different middle schools and one high school	40 minutes presentation, presented in a series of sequential steps. Follow-up reported at six weeks	Increased middle school and high school students' knowledge Of the effects of alcohol consumption during pregnancy.
LaChausse, 2008	USA	Longitudinal quasi-	114 culturally diverse youth	Teen peer educators used the Fetal Alcohol Spectrum Teaching and Research Awareness	The program increased participants' knowledge regarding FAS but had no

		experimental design (two groups)		Campaign (FASTRAC) to teach their fellow students. The FASTRAC intervention solely consisted of this 45-minute PowerPoint presentation. No additional learning activities or materials were used. Follow up reported at 1 week	significant effect on participants' attitudes, beliefs about the dangers of FASD or intention to use alcohol during pregnancy.
**Social**					
De Vries et al., 2013	South Africa	Prospective study	67 women (pregnant women) between ages 18 to 40 years who are at high risk for bearing a child with FASD	Self-Administered Questionnaire (SAQ) and the Alcohol Use Disorders Identification Test (AUDIT) were used to assess alcohol use. Follow up reported at 6, 12, and 18 months respectively	Alcohol consumption drops significantly when compared before pregnancy to the second and third trimesters
Montag et al., 2015	USA	Randomized control trial	263 women American of Indian/Alaska Native (AIAN) descents were recruited	Baseline, web-based and follow up questionnaires were used to access information on birth control use and alcohol consumption. Follow up reported at 1, 2, and 6 months	Randomization to the SBIRT did not result in a significantly different change in risky drinking behaviors
May et al., 2103	South Africa	Prospective study	41 women (pregnant women) between ages 18 to 40 years who are at high risk for bearing a child with FASD	The Alcohol Use Disorders Identification Test (AUDIT) was used to access the drinking problem. The Happiness Scale and Psychological Pain were used to access the participants' mental health. Blood Alcohol Concentrations (BAC) were estimated by the BACCUS technique. Follow up reported at 6, 12, and 18 months respectively.	Case management was particularly valuable for pregnant women, as statistically significant reductions in alcohol risk were obtained.
Wilton et al., 2013	USA	Randomized control trial	Women between the ages of 18 and 44 who were drinking above recommended levels	A timeline follow-back (TLFB) methodology was used to document the alcohol consumption and sexual intercourse of participating women. Follow up reported at 6 months	Telephone-based brief intervention may be equally successful and cost-effective in reducing the risk of an alcohol-exposed pregnancy and thus fetal alcohol syndrome.
Rasmussen et al., 2012	Canada	Retrospective analysis	70 women between the ages of 18 and 41	Clients were then assigned a program mentor who began to build a relationship with the client. Mentors collected additional intake assessment data within the first few weeks of the program (including Needs Assessments	The First Steps program demonstrated promising outcomes for women at-risk for giving birth to a child with FASD

				and Goals Assessments). Follow up reported up to 28 months	
Floyd et al., 2007	USA	Randomized control trial	830 non-pregnant women, aged 18–44 years	The intervention was delivered over 14 weeks, with approximately 2 to 3 weeks between sessions. The counseling sessions and the contraception consultation visit were each 45 to 60 minutes.	Brief motivational intervention can reduce the risk of an AEP.
Hanson et al., 2012	USA	Survey	119 American Indian women of childbearing age (18–44)	The participants were asked to respond to the Likert-scale questions regarding the media campaign. No follow-up Reported	The finding indicated that the campaign decreases the participants' drinking behaviors.
Hanson et al., 2017	USA	Pre-post-test design	A total of 193 non-pregnant American Indian women enrolled in the OST CHOICES Program, and all were at risk for AEP because of binge drinking and being at risk for unintended pregnancy.	Interventionists provided 2 or 4 CHOICES sessions, per the preference of the site and stakeholder input. The sessions were held approximately 1 to 2 weeks apart. Follow up reported at 3 and 6 months	Findings showed a significant decrease in AEP risk from baseline at both 3- and 6-month follow-ups.
Letourneau et al., 2017	USA	Dichotomized control trial	89 women who identified as Hispanic between the ages of 18–46 years	The Timeline Followback (TLFB) was used to assess daily drinking and<br>The Quick Drinking Screen (QDS) was used to measure alcohol use and collect aggregate drinking data.<br>Follow up reported at 6 months	The finding showed at the 6-month follow-up, two thirds (66%) of all Hispanic women had reduced their overall risk of an AEP, primarily by practicing effective birth control.
O'Connor and Whaley, 2007	USA	Randomized control trial	345 pregnant women who were participants in the Public Health Foundation Enterprises Management Solutions Special Supplemental Nutrition Program for Women, Infants, and Children	Participants received brief intervention consisted of 10- to 15-minute sessions of counseling by a nutritionist, who used a scripted manual to guide the intervention. Follow up reported at 3rd trimester of the pregnancy.	Increased individual's motivation to change unhealthy behavior.
Hanson et al., 2013	USA	Descriptive longitudinal study	231 non-pregnant American Indian women	Participants responded to drinking and contraception questions through the telephone and then received intervention materials via mail. Follow up reported at 3, 6, 9, and 12 months	The intervention was successful in modifying self-reported drinking and contraception behaviors.

Farrell-Carnahan et al., 2013	USA	Prospective	Women between the ages of 18 and 44 years who drink and who also have sex	The intervention was conducted during one 60-min telephone call and consisted of the EARLY intervention translated into a mail and telephone-administered format.<br>Follow up reported at 3 and 6 months	Remote delivery of intervention was feasible, and the translated remote intervention may reduce AEP risk
France et al., 2014	Australia	Randomized control trial	354 women of childbearing age who were not pregnant (n = 354) and 116 pregnant women	Intentions and confidence were measured by single-item questions that were measured on a five-point scale. The AUDIT-C alcohol screening tool was used to access the drinking problem.<br>No follow Up reported	Finding provides important insights into the components that enhance the persuasiveness and effectiveness of messages aimed at preventing prenatal alcohol exposure
Dresser et al., 2011	USA	Pre-post-test (two groups)	Experimental (n = 148) and comparison (n = 183) licensed alcohol establishments staff. 1330 alcohol servers were provided comprehensive FAS prevention training	Each person attending the 2-hour training session received a stipend and a laminated card with FAS facts and strategies for servers. Follow up reported at 1, 6, and 12 months	Supplemental responsible beverage service training for alcohol can be effective in reducing the serving of alcohol to visibly pregnant women.
Chersich et al., 2012	South Africa	Pre-post-test design	809 children were enrolled	A pamphlet and poster were designed and distributed everywhere in the community. Regular articles focusing on FASD prevention were published in local community newspapers. Follow up reported at 9 and 18 months	Universal prevention might reduce FASD by ~30% and have population-level effects.
Tenkku et al., 2011	USA	Pre-post intervention	458 women between the ages of 18 and 44 who were at risk for an AEP were eligible for participation	The intervention was designed using tailored motivational messaging and consisted of four modules—Where Am I Now, Decisional Analysis, Goal Setting and Planning, and Overcoming Barriers.<br>Follow up reported at 4 months	Mail and online versions of the intervention were equally successful at reducing the risk for an AEP
			**Management intervention**		
**Clinical**					
Nguyen et al., 2016	USA	Randomized, double-blind, placebo-controlled clinical trial	55 children between age 5 to 10years participated. Choline (n = 29) or placebo (n = 26) treatment arms.	Participants in the choline group received 625 mg choline/d for 6 weeks, whereas subjects in the placebo group received an equivalent dose of an inactive placebo treatment. No follow-up reported.	The finding shows the current study does not support that choline, administered at a dose of 625 mg/d for 6 weeks, is an effective intervention for

					school-aged (5–10 years old) children with FASD.
Zarnegar et al., 2016	USA	Pre-post-test design	Children's ages ranged from 10 to 53 months.<br>Caregivers' ages were between 32 and 58 years	Children and caregivers received Child-Parent Psychotherapy (CPP), and caregivers also received Mindful Parenting Education (MPE). Follow-up reported at 6 month	Findings show that children's adaptive, motor, communication, and cognitive skills have improved in a short amount of time.
Wozniak et al., 2015	USA	Double-blind, randomized, placebo-controlled.	Children (2.5–5 years at enrollment) with FASD (n = 60) who received 500 mg choline or a placebo daily for 9 months	Children with FASD (n = 60) who received 500 mg choline or a placebo daily for 9 months. Outcome measures were Mullen Scales of Early Learning (primary) and the elicited imitation (EI) memory paradigm. No follow-up reported.	Finding shows that the administration proved feasible, and choline was well tolerated.
Wozniak et al., 2013	USA	Double-blind, randomized, placebo-controlled.	20 children ages 2.5–4.9years Participants were randomly assigned to 500 mg. choline or placebo daily for nine months	Participants were randomly assigned to 500 mg. choline or placebo daily for nine months (10 active; 10 placebo. No follow-up reported.	The finding shows that choline supplementation at 500 mg per day for nine months in children ages 2–5 is feasible and has high tolerability
Wilczynski et al., 2015	Poland	Pre-post-test design	20 children (14 boys, 6 girls) aged 4–5 years	9 school-term months of treatment, general scholastic tests, teacher and parent questionnaires, classroom observations and specific language and literacy tests were administered to the participants. No follow-up reported.	The finding shows significant cognitive improvements in specific areas targeted by classroom interventions
Yazdani et al., 2009	Canada	Dichotomized control trial	Mother with alcohol used during pregnancy (n=28) and Mother without alcohol used during pregnancy (n = 10)	Children were tested on either the BSID-III or WPPSI-III assessment tool. No follow-up reported	This pilot suggests that early intervention may mitigate some of the well-described damages caused by heavy in utero alcohol exposure.
Kable et al., 2015	Ukraine	Randomized controlled trial	Women of moderate to heavy drinking (n = 301) and low/unexposed (N = 313)	Alcohol-using and nondrinking women were randomized to one of three multivitamin/mineral supplement groups: none, multivitamins/minerals (MVM), and multivitamin/minerals plus choline. Children (N = 367) were tested at 6 months with the Bayley Scales of Infant Development (2nd ED. Follow-up reported at 6 months	Multivitamin/mineral supplementation can reduce the negative impact of alcohol use during pregnancy on specific developmental outcomes

Connolly et al., 2016	USA	Case study	One child (3 years 10 months)	The child received 15-hour of intensive one-on-one applied behavior analysis (ABA) therapy each week for 23 months. Reported follow-up at 9 month	Finding the indicated rapid skill acquisition across several areas of functioning.
Nash et al., 2015	Canada	Randomized control trial	Twenty-five children aged 8–12 years diagnosed with an FASD were assigned in alternating sequence to either an immediate treatment (TXT) or a delayed treatment control (DTC) group	Children received 12 one hour sessions over 14weeks treatment specifically designed to target self-regulation, a component of executive functions (EF). Reported follow up at 6 months.	The result shows EF disabilities in children with FASD can be remediated through a targeted treatment approach aimed at facilitating self-regulation skills.
**Educational**					
Coles et al., 2009	USA	Randomized control trial	Children between the age of 3 to 10 years with FASD, their caregivers and teachers participated in the programme. 87 children were recruited, 61 children participated in the intervention, 56 children completed study 1 post testing and 54 children participated in the study 2 post testing	The average duration between Pre-test and Post Test 2 was about 13 months For mathematics, intervention families were randomized to either the math intervention group or the standard psychoeducational treatment contrast. Standard psychoeducational treatment consisted of a comprehensive neurodevelopmental evaluation and assistance with educational placement and development of the individualized educational plan within the context of their home school. In addition to these services, those in the math intervention group received 6-weeks of tutoring services. Follow-up reported at 6 months	Findings show that the current study provides evidence that the initial results were not transitory as might be a concern for such a short-term intervention. And, it appears that providing parents with appropriate tools for working with their children can result in significant improvement in child behavior in both home and school.
Kerns et al., 2010	Canada	Quasi-experimental design	12 students were identified and enrolled in the study. The final sample was comprised of 10 children (six males, four females) Two dropped out.	The intervention program was delivered via a laptop computer for adopted parents and teachers. They returned the short follow-up evaluation questionnaire. No follow-up reported.	Results show improvement in cognitive performance in children with FASD on measures of distractibility, sustained attention, divided attention, working memory, math, and reading.
Jirikowic et al., 2016	USA	Randomized control trial	29 children with FASDs, aged 8 years to 15 years 8 months	Children with FASD received STABEL training in a university laboratory, or home, or were controls. The Movement Assessment Battery for Children–2nd edition (MABC-2) and Pediatric Clinical Test of Sensory Interaction	Preliminary results support improved sensory adaptation, balance, and motor performance.

				for Balance–2 (P-CTSIB-2) were analyzed by group (lab, home, and control), session (pre-STABEL, 1-week post-STABEL, and 1-month post-STABEL), and group-by-session interaction. Reported follow-up after 1 week and 1 month	
Adnams et al., 2007	South Africa	Randomized control trial	65 third grade, nine-year-old children	9 school-term months of treatment, general scholastic tests, teacher and parent questionnaires, classroom observations and specific language and literacy tests were administered to the participants. No follow up reported	The finding shows significant cognitive improvements in specific areas targeted by classroom interventions.
Kable et al., 2007	USA	Pre-post-test design	61 children 3 to 10 years of age	Children received a neurodevelopmental evaluation to assist with their educational planning. Testing was done over 2 days, requiring approximately 4 to 5 hours to complete. No follow-up reported.	Finding reveals psych educational the program may help to remediate deficits associated with prenatal alcohol exposure.
Kable et al., 2016	USA	Randomized control trial	Children (n = 30), ages 5 to 10, with FASD were recruited and randomly assigned to one of three groups.	Children assigned to one of the two intervention groups attended 5, 1-hour long individualized computer instructional sessions while their parents were being trained. No follow-up reported.	The result showed that children who received computerized instruction consistent with the parent training demonstrated greater self-regulation improvements than those receiving incongruent computerized instruction
McCoy et al., 2015		Pre- and post-test design	Eleven children with FASD and 11 children with TD, aged 8 to 16 years	Children completed 30 minutes of STABEL training. The children answered questions about their experience using STABEL. No follow Up reported.	Children with FASD showed higher entrainment gain to vestibular stimuli
Kerns et al., 2017	Canada	Pre- and post-test	10 Children (ages 6–13)	Game-based process specific intervention as a potentially effective treatment and useful tool for supporting cognitive improvements in children with FASDs. No follow-up reported	EA provides children with instruction in metacognitive strategies to improve gameplay, with participants completing approximately 12 hours of training over an 8 to 12 school week period.
Coles et al., 2015	USA	Randomized control trial	30 children between the ages of 5 to 10 years	Volunteer families were randomly assigned to 1 of 3 groups: (i) GoFAR; (ii) FACELAND; or (iii) CONTROL. GoFAR group received instruction via a computer program that was	Result suggests that GoFAR game supported positive behavior change and effective in reducing disruptive behaviors.

				designed specifically for the intervention, the FACELAND group received instruction via a computer program, and the control group received no interventions. No report on the long-term effect of the intervention.	
Wells et al., 2012	USA	Randomized control trial	78 children between 6 to 11.9 years participated in the program. 40 children in the treatment group and 38 in the control	Caregiver and child groups lasted approximately 75 minutes and were conducted concurrently by doctoral- and masters-level therapists, including an occupational therapist for 12 weeks. The Behaviour Rating Inventory of Executive Function was used to measure indicators for executive functioning. The Roberts Apperception Test for Children was used to measure adaptive and maladaptive functioning Follow-up measures were administered 7 months after enrolment.	The findings show improving executive functioning and emotional problem-solving in children with FAS or ARND.
Keil et al., 2010	USA	Randomized control trial	100 children (51% male) with PAE between the ages of 6 and 12 years	The CFT group received 12 sessions, of 90 minutes in length, delivered over the course of 12 weeks. 3-month follow-up reported.	Social skills intervention improves deficits in social information-processing among individuals with PAE.
O'Connor et al., 2016	USA	Randomized control trial	54 adolescents participated. 18 were classified moderate/light drinkers and 36 were classified as abstinent/infrequent drinkers	The intervention consisted of 6, 60-minute sessions delivered over the course of 6 weeks. Caregivers and adolescents attended separate but concurrent sessions. Reported follow-up at 3 months.	The finding shows a reduction in alcohol use and its negative consequences in adolescents with FASD who were more experienced in the use of alcohol.
Schonfeld et al., 2009	USA	Randomized control trial	100 children between the ages of 6 and 12	Children in the CFT group received 12 sessions, 90 minutes in length, delivered over the course of 12 weeks. Following a 12-week waiting period, children in the DTC group received treatment identical to the CFT group. No follow Up reported.	The objective was achieved. Finding shows the ability to control impulses, solve problems flexibly, and monitor emotional responses significantly predicted improvement in social skills and reduction in problem behaviors following Children's Friendship Training (CFT)
**Social**					
Leenaars et al., 2012	USA	Retrospective case analysis	186 families parenting at least one child with FASDs.	Participants rate Needs and Goals and Stress Scale and Client Satisfaction Surveys. No follow up reported	Result reveals a significant decrease in caregiver stress from pre- to post-program a significant decrease in needs

			Mean age was 46 years (Range 24–69 years). 30% of the caregivers were foster parents, 23% adoptive, 19% biological mother or father, 15% kinship or a biological relative, and 9% were permanent guardianship order		and increase in goal attainment from pre-to post-program
O'Connor et al., 2012	USA	Randomized control trial	Of the 85 children were recruited 41 were assigned to the CFT condition and 44 were assigned to the SOC condition. Of that number, 67 children (CFT, n = 32; SOC, n = 35) completed the 12-week study	Both the CFT and the SOC conditions consisted of 12 sessions, of 90 minutes each, delivered over the course of 12 weeks.<br>No follow Up reported.	The result showed children participating in CFT showed significantly improved knowledge of appropriate social skills, improved self-concept, and improvements in parent-reported social skills compared to children in the SOC condition.
Petrenko et al., 2017	USA	Randomized control trial	56 children (6–13 years) with FASD	EU was measured by the Kusche Affective Inventory-Revised (KAI-R25), as part of a larger test battery.<br>Follow up reported at 17. 64 months	Treatments targeting emotional understanding (EU) may benefit children with FASD as components within a comprehensive, tailored intervention focused on child self-regulation and caregiver behavior management.
Reid et al., 2017	Australia	Mixed method	Three families with FASD children. Age ranges from 9 to 12 years	Families attended 1 to 2 hours weekly or fortnightly session. 86-item Behaviour Rating Inventory of Executive Function was used to evaluate Children's executive functioning abilities in their daily activities. The 64-item Youth Outcome Questionnaire –parent report was used as a repeated measure to evaluate the children's psychosocial distress.<br>Parents were an interview.<br>Follow up was reported at 3 months	The result provides preliminary support for the feasibility of an adapted version of the PuP program.
Petrenko et al., 2017	USA	Randomized control trial	30 children with FASD or prenatal alcohol exposure (PAE) (ages 4 to 8) and their primary caregivers were enrolled.	The 30-week intervention integrates scientifically validated bimonthly, in-home parent behavioral consultation, and weekly child skills groups. Follow-up reported at 9 months	Findings suggest that the Families on Track Program had the largest impact on the child-related outcomes of emotion regulation and self-esteem

Millians and Coles, 2014	USA	Case Study	Five children between the ages of 10 to 13 years. With FASD or suspected participated in the program	The program occurred for 20 Saturdays from September 2005 through May 2006. All of the children were administered the Matrix Analogies Test, Expanded Form (MAT: EF) Two children were administered the Test of Reading Comprehension, Third Edition (TORC-3). The Test of Word Reading Efficiency (TOWRE) was administered One child was administered the Gray Oral Reading Test, Fourth Edition. One Wechsler Individual Achievement Test, Second Edition (WIAT-II). Reported follow-up at 6months.	The program showed clinically significant gains in learning and academics.
Kable et al., 2015	USA	Randomized control trial	60 participants	60 participants were randomly assigned to one of the three treatment groups: the MILE program administered at a specialty care center (Center MILE) or in the community (Community MILE), or to parent math instruction only (Parent Instruction). Children received a neurodevelopmental evaluation to assist with their educational planning. Testing was done over 2 days, requiring approximately 4 to 5 hours to complete.	The finding shows that MILE was well-received and effective in producing positive treatment outcomes.
Pomeroy and Parrish, 2013	USA	Pre- and post-test design	338 CASA volunteers and staff from 55 CASA locations in Texas,	A three-hour online FASD training on CASA workers' knowledge of FASD and their comfort and confidence in identifying children with FASD for referral, advocating for them, and linking. No follow up reported	The results support the potential of this online training to enhance CASA volunteers' ability to help children with FASD
Hanlon-Dearman et al., 2017	Canada	Randomized controlled trial.	Twelve caregiver-child dyads completed the FASD adapted COS intervention. Children's ages ranged from 2–5 years	The family received behavioural assessment and interventions focused on education regarding PAE related challenges, as well as proactive and reactive behavioral intervention strategies. Follow-up reported at 3 months.	Parents also showed an improvement in their ability to attend to their child's cues
